# Impaired Fracture Healing after Hemorrhagic Shock

**DOI:** 10.1155/2015/132451

**Published:** 2015-04-01

**Authors:** Philipp Lichte, Philipp Kobbe, Roman Pfeifer, Graeme C. Campbell, Rainer Beckmann, Mersedeh Tohidnezhad, Christian Bergmann, Mamed Kadyrov, Horst Fischer, Christian C. Glüer, Frank Hildebrand, Hans-Christoph Pape, Thomas Pufe

**Affiliations:** ^1^Harald Tscherne Research Laboratory for Orthopedic Trauma, Department of Orthopaedic Trauma Surgery, University Hospital RWTH Aachen, Pauwelsstraße 30, 52074 Aachen, Germany; ^2^Department of Orthopaedic Trauma Surgery, University Hospital RWTH Aachen, Pauwelsstraße 30, 52074 Aachen, Germany; ^3^Department of Radiology, Molecular Imaging North Competence Center (MOIN CC), University Hospital Schleswig-Holstein, Campus Kiel, Am Botanischen Garten 14, 24118 Kiel, Germany; ^4^Institute of Anatomy and Cell Biology, RWTH Aachen University, Wendlingweg 2, 52074 Aachen, Germany; ^5^Department of Dental Materials and Biomaterials Research, University Hospital RWTH Aachen, Pauwelsstraße 30, 52074 Aachen, Germany

## Abstract

Impaired fracture healing can occur in severely injured patients with hemorrhagic shock due to decreased soft tissue perfusion after trauma. We investigated the effects of fracture healing in a standardized pressure controlled hemorrhagic shock model in mice, to test the hypothesis that bleeding is relevant in the bone healing response. Male C57/BL6 mice were subjected to a closed femoral shaft fracture stabilized by intramedullary nailing. One group was additionally subjected to pressure controlled hemorrhagic shock (HS, mean arterial pressure (MAP) of 35 mmHg for 90 minutes). Serum cytokines (IL-6, KC, MCP-1, and TNF-*α*) were analyzed 6 hours after shock. Fracture healing was assessed 21 days after fracture. Hemorrhagic shock is associated with a significant increase in serum inflammatory cytokines in the early phase. Histologic analysis demonstrated a significantly decreased number of osteoclasts, a decrease in bone quality, and more cartilage islands after hemorrhagic shock. *μ*CT analysis showed a trend towards decreased bone tissue mineral density in the HS group. Mechanical testing revealed no difference in tensile failure. Our results suggest a delay in fracture healing after hemorrhagic shock. This may be due to significantly diminished osteoclast recruitment. The exact mechanisms should be studied further, particularly during earlier stages of fracture healing.

## 1. Introduction

Bone fractures are a significant clinical and economic problem. The majority of fractures restore original structure and function in a scarless manner. Different reasons might be responsible for delayed or nonunion healing [1]. In the clinical setting, fracture healing is influenced by factors involving additional injuries [2]. Delayed or nonunion increases the cost of care, necessitates additional surgeries, and results in a prolonged period of convalescence, which is associated with increased mortality [3]. The incidence of hemorrhagic shock following long bone fractures is common among severely injured patients. Fracture healing is a complex interaction of mechanical, cellular, and biological pathways. The inflammatory response after trauma may also represent a significant factor in bone healing by regulating enchondral bone formation and bone remodeling [4, 5]. Therefore, the immune response after hemorrhagic shock involves a complex interaction characterized by the release of diverse inflammatory mediators (e.g., cytokines) and a vast array of immune competent cells into organ tissue. However, the results of experimental studies investigating fracture healing in models with additional trauma are controversial. Some studies identified salutary effects of an additional traumatic insult [6, 7], whereas others observed a negative influence of trauma-hemorrhage on fracture healing [8]. However, these studies utilized significantly different hemorrhagic shock protocols, which may contribute to the controversial results.

We investigated the effect of hemorrhagic shock on fracture healing in a standardized mouse model of a closed femoral fracture in combination with pressure controlled hemorrhagic shock in order to test the hypothesis that hemorrhage impairs bone healing.

## 2. Material and Methods

### 2.1. Animal Care

All animal studies were carried out in accordance with the German Animal Welfare Legislation, and experiments were permitted by the local government of North Rhine-Westphalia, Germany.

Male C57/BL6 mice weighing 25.0 ± 2.0 g were obtained from Charles River Laboratories (Sulzfeld, Germany). Animals were maintained under standardized conditions and environment. During the study period, all mice were kept by a 12-hour light-dark cycle and a constant room temperature. Water and pelleted chow were available ad libitum on the ground of the cage. Analgesia was administrated by subcutaneous Buprenorphine 0.1 mg/kg twice a day.

All procedures were performed under deep anesthesia with 50 mg/kg intraperitoneally injected phenobarbital and inhaled isoflurane. After completion of surgery, the mice were warmed with warming mats.

### 2.2. Group Distribution

Animals were randomly assigned to one of two experimental groups, with each group consisting of 14 animals. Animals of the control group received a femoral fracture on the right side and implantation of the catheter into the left femoral artery but did not sustain blood loss (group Fx), while the experimental group included animals with a right sided femoral fracture and pressure controlled hemorrhagic shock (group FxHS).

### 2.3. Induction of Hemorrhagic Shock

The left femoral artery was cannulated with polyethylene tubing (Becton Dickinson, Sparks, MD). Blood pressure was measured via the arterial line using a blood pressure analyzer (Micro-Med, Louisville, KY, USA). Animals were bled through an arterial catheter to a mean arterial blood pressure of 35 ± 5 mmHg within 5 min, which was then maintained for 90 minutes. Afterwards, the animals were resuscitated with two times the shed blood volume using Ringer's solution (Berlin-Chemie-AG, Berlin, Germany). After removing the catheter, the wound was closed with sutures.

### 2.4. Induction of Femoral Fracture

The right femur was stabilized by intramedullary fixation prior to fracture induction. Retrograde nailing was performed through a small incision lateral to the patella. Blunt dissection was used to expose the femoral notch and a 27G needle was introduced into the intramedullary canal through the proximal metaphyseal zone as described by Bonnarens and Einhorn [9]. The cannula was then shortened beneath the cartilaginous surface. The wound was closed by simple interrupted sutures. A transverse femoral fracture was induced by a standardized blunt guillotine device.

### 2.5. Assessment of TNF*α*, IL-6, MCP-1, and KC Plasma Concentrations

50 *μ*L of blood was taken two hours before and six hours after fracture induction by retrobulbar venous puncture. Heparinized blood was centrifuged for 10 min at 5000 rpm at 10°C. Plasma was separated and stored at −80°C. Concentrations of TNF*α*, IL-6, MCP-1, and KC were measured by Bio-Plex Pro assays (Biorad, Hercules, CA, USA) according to the manufacturer's instructions.

### 2.6. Harvesting Procedure

Animals were anesthetized with isoflurane 21 days after fracture and euthanasia was performed by cervical dislocation. The femoral bones were harvested and seven of each group were fixated in formalin for histological examination. Both femora of the other seven mice were frozen for *μ*CT examination and mechanical testing.

### 2.7. Histology

Tissue samples were decalcified in 12.5% EDTA (Schweizerhall Chemie AG, Basel Switzerland) with 1.25%  sodium hydroxide (Fluka) as confirmed by radiography and embedded in paraffin. Sections of 4–6 micrometers were cut longitudinally through the center of the medullary canal using a Leica microtome. They were placed onto Histobond microscope slides (Marienfeld, Germany) and left overnight at 37°C. Sections were routinely stained according to Drescher et al. [10].

Osteoclasts were detected by staining of tartrate resistant acid phosphatase (TRAP). The sections were pretreated with TRAP-buffer (40 mM sodium acetate (Merck), 200 mM sodium tartrate dihydrate (Merck) in aqua dest., pH5) for 10 minutes followed by incubation in a staining-solution (19,4% (w/v) naphthol AS-phosphate mix (Sigma), 116,5% (w/v) Fast Red Violet LB salt (Sigma), Triton-X100 (Sigma) 1% (v/v), and 1,9% (v/v) N-N-dimethylformamide (Sigma) solved in TRAP-buffer) at 37°C for 2 hours. After washing in aqua dest., the slides were counterstained with methyl green.

The fracture regions were examined using light microscopy at a magnification of 200x. The analyses were performed by 2 blinded examiners (RB and MK) who were unaware of the treatment. Three fields were randomly assigned in the field of interest (callous area). The number of TRAP-positive cells and cartilage segments in these fields were counted.

The bone quality was assessed using the score system: score 1, only connective tissue and cartilage within the callus area; score 2, woven bone with some islands of cartilage and connective tissue; score 3, woven bone without any cartilage at the callus area; score 4, woven and lamellar bone at the callus area; score 5, only lamellar bone at the callus area.

### 2.8. Micro-CT Analysis

The entire callus region in each sample was micro-CT scanned (vivaCT 40, Scanco Medical, Brüttisellen, Switzerland) with an isotropic voxel size of 25 *μ*m (55 kVp, 145 *μ*A, 200 ms integration time, 500 projections on 180° 1024 CCD detector array, and cone-beam reconstruction). The accuracy of the scanner was monitored weekly for density measurements using hydroxyapatite (HA) phantoms with densities of 0, 100, 200, 400, and 800 mg HA/cm^3^ and monthly for geometry measurements using aluminum rods with a known volume of 0.104 mm^3^.

In the reconstructed micro-CT images, a semiautomated contouring method was used to outline the inner and outer boundaries of the callus structure. The volume within the contour was then isolated, and the volumetric bone mineral density (BMD, bone mineral content divided by the entire analyzed volume) was determined from the grayscale image (Image Processing Language v5.15, Scanco Medical Switzerland). The images were Gaussian filtered (*σ* = 0.8, supp = 1) and thresholded (19% of maximal grayscale value) resulting in binarized images containing only bone and background. The bone volume ratio (BV/TV) was then determined as the ratio of bone tissue divided by the entire volume and the tissue mineral density (TMD) as the bone mineral content within the bone voxels divided by the total volume of bone voxels.

### 2.9. Mechanical Testing

The tensile strength of mouse femora was analyzed using a universal testing machine (Z2.5, Zwick, Ulm) with a 200 N load cell. The mouse femora were fixed at the proximal and distal ends by alligator clips. The alligator clips were attached to cardan joints and the construct was placed into the universal testing machine. The cardan joints were used to prevent angular mounting and to ensure straight tensile load along the femoral shaft. The mechanical testing was performed with no initial load and a constant feed rate of 1 mm/min. The femora were tested until total fracture and the strength were measured.

### 2.10. Statistical Analysis

Statistical analyses were performed using SPSS software (SPSS Inc., Chicago, IL, USA). Results are presented as means ± SEM. In normally distributed variables, group comparisons were assessed using ANOVA followed by Tukey's HSD test. Nonnormally distributed parameters were tested using the Kruskal-Wallis test. The null hypothesis was rejected for *P* < 0.05.

## 3. Results

Overall 32 animals were included in this study. Two mice died during haemorrhagic shock, one mouse was euthanized due to failure of osteosynthesis, and one mouse died on day 10. Both study groups consist of 14 mice at the endpoint at day 21.

**Figure 1:**
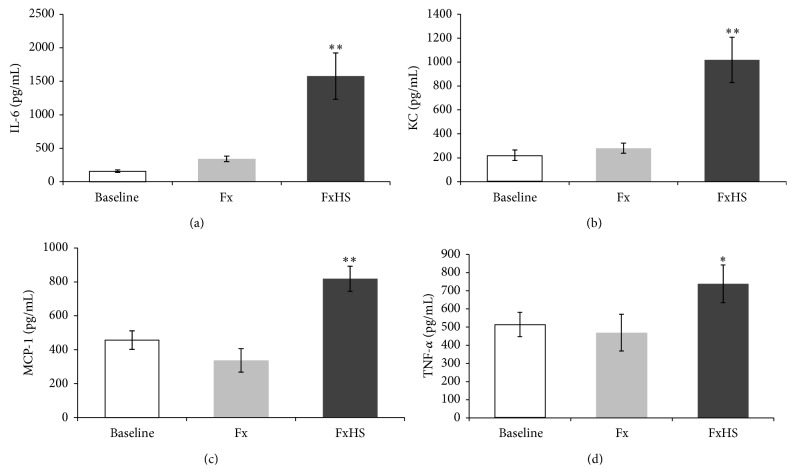
Concentration of plasma cytokines IL-6 (a), KC (b), MCP-1 (c), and TNF-*α* (d). Baseline measurement was 2 hs before and Fx and FxHS 6 hs after shock. Results are presented as mean ± SD. ^*^ *P* < 0.05; ^**^ *P* < 0.001 in comparison to Fx.

We measured the proinflammatory cytokines IL-6, MCP-1, KC, and TNF-*α* at baseline and 6 hours after operation. Each cytokine demonstrated significant increases in group FxHS compared to the Fx group and to the baseline values (Figures 1(a)–1(d)). IL-6 showed no significant difference between the baseline value and Fx group (156.6 versus 340.4 pg/mL; *P* = 0.21) but a nearly fivefold increase between Fx and FxHS (340.4 versus 1577.3 pg/mL; *P* < 0.001) (Figure 1(a)). The increase in KC serum levels in group FxHS was also nearly fourfold in comparison to group Fx (280.0 versus 1018.7 pg/mL; *P* = 0.002) (Figure 1(b)). MCP-1 and TNF-*α* also demonstrated a significant difference between the FxHS and Fx groups (MCP-1: 336.2 versus 818.1; *P* = 0.002, TNF-*α*: 469.4 versus 738.5; *P* = 0.001) (Figures 1(c) and 1(d)). Baseline and Fx values were comparable regarding the measured cytokines.

**Figure 2:**
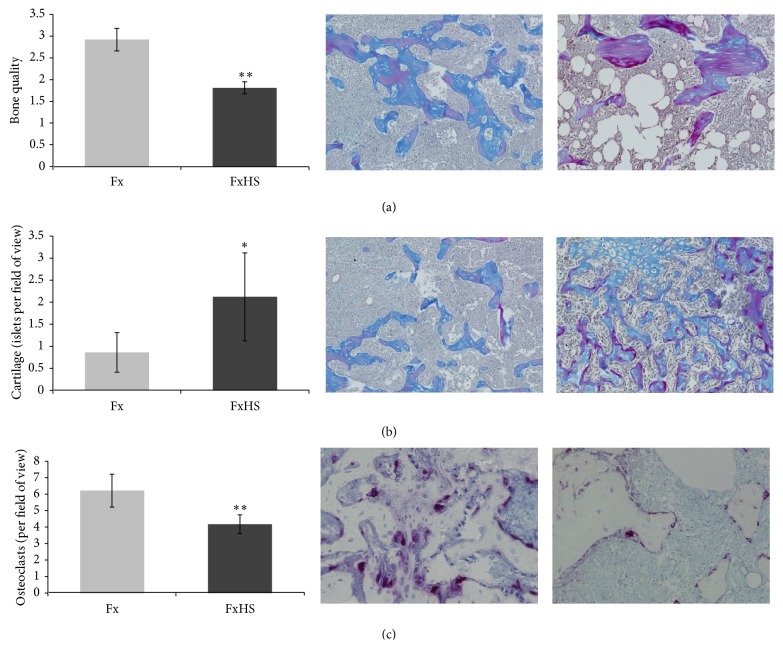
Histological analysis. Specimens were blinded and scored by two independent observers. Pictures are representative for the 7 slides per group. Results are presented as mean ± SD. ^*^ *P* < 0.05; ^**^ *P* < 0.001 in comparison to Fx.

Blinded histological evaluation by two independent experts using the above-mentioned score from 1 to 5 showed a significant decreased bone quality in the FxHS group (2.9 versus 1.8; *P* = 0.001). In parallel we could identify more cartilage segments in the callus region (0.9 versus 2.1; *P* = 0.002) and the number of osteoclasts was significantly decreased in FxHS (6.2 versus 4.2; *P* = 0.001) (Figures 2(a)–2(c)) compared to fractures without HS (Fx).

**Figure 3:**
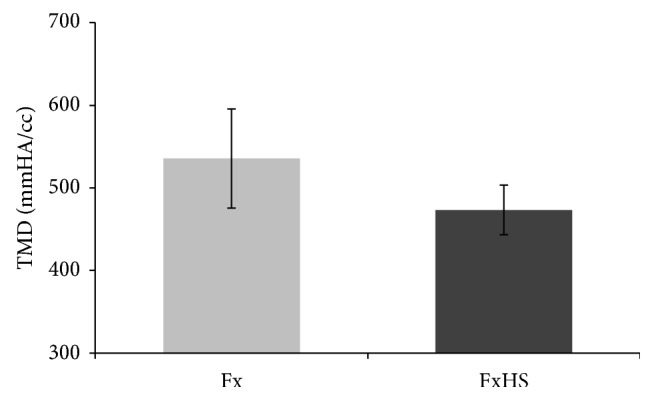
*μ*CT measurements showed a trend towards a decreased mineral density in the FxHS group (*P* = 0.098). Results are presented as means.

**Figure 4:**
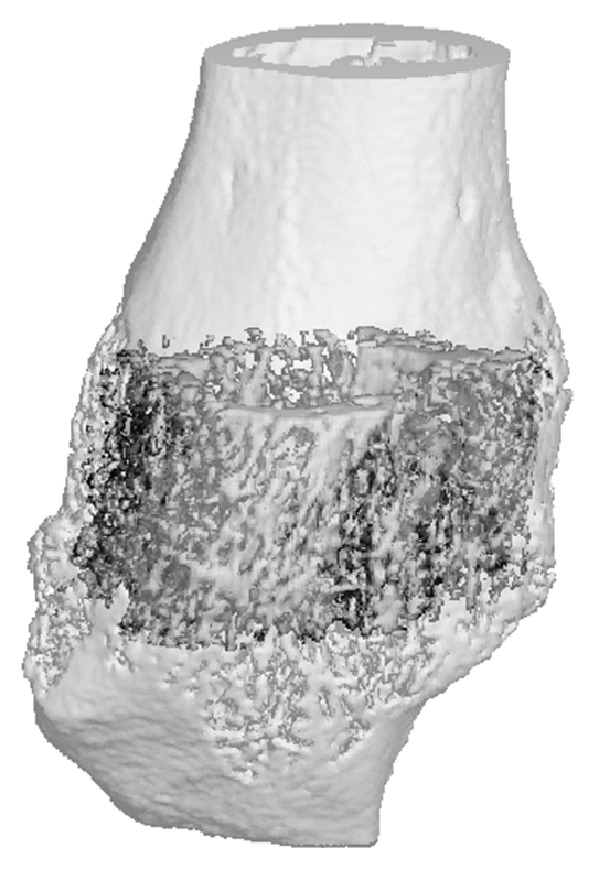
Hybrid *μ*-CT image of the fracture region. The callous region is colored darker.

Micro-CT analysis (Figure 4) showed no significant differences in BMD or BV/TV between Fx and FxHS. While not statistically significant, there was a trend towards reduced TMD in the FxHS group compared to the Fx group (*P* = 0.098) (Figure 3).

**Figure 5:**
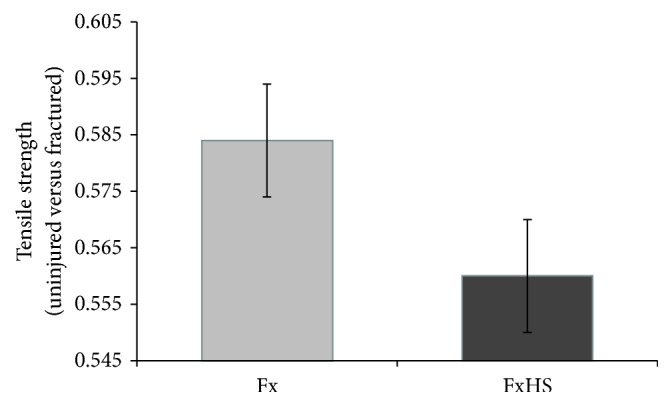
Tensile strength is presented as means of the quotient of the fracture side divided by the opposite femur.

As expected, the tensile strength of the fractured femora did not reach the strength of the uninjured opposite side. However, comparison of the relative failure strength (fractured right femur/uninjured left femur) between groups FxHS und Fx showed no significant difference (0.58 versus 0.56; *P* = 0.71) (Figure 5).

## 4. Discussion

We analyzed hemorrhagic shock-related differences in fracture healing in a femoral fracture model in mice. Our main findings are as follows.

1. We could observe a typical inflammatory response after FxHS whereas Fx alone caused no significant increase in inflammatory cytokines.
2. Histological examination three weeks after fracture showed significantly decreased number of osteoclasts and reduced bone quality after hemorrhagic shock. In parallel, *μ*CT analyses showed a trend towards decreased mineral density in the fracture region after hemorrhagic shock. Despite these microscopic findings, the mechanical properties of the fractured femora were not affected by hemorrhagic shock.

Three major factors are crucial for sufficient fracture healing: stability, blood supply, and the presence of osteogenic mediators. The need of mechanical stability is already sufficiently discussed in the literature. It is known that interfragmentary shearing forces and movement can delay fracture healing or lead to nonunions [11–13].

A sufficient blood supply is predicated on the integrity of the vascular system and on the circulating blood volume. Disorders in angiogenesis have a negative influence on fracture healing especially in the early stages after fracture [14, 15]. Bumann et al. reported no changes in the blood supply in the fracture region in rats after hemorrhagic shock [7]. Furthermore, the mechanical properties of the fracture were not affected four weeks after injury. These results confirmed the results of Lucas et al. who showed an osteogenic effect of hemorrhagic shock in rats [6]. Additionally, a study in goats revealed no differences in mechanical properties, bone formation rate, and callus remodeling after hemorrhagic shock [16].

The third factor in fracture healing is the release of osteogenic mediators. Recent studies have focused on osteoimmunology, specifically the importance of cellular and molecular interactions between the immune system and the bone. In addition, T-lymphocytes [17] and cytokines seem to hold specific relevance in fracture healing in the context of osteogenic mediators [18, 19]. The proinflammatory cytokine IL-6 is involved in the regulation of the differentiation of osteoclast progenitor cell into mature osteoclasts [20, 21]. On the other hand, IL-6 can suppress chondrocyte proliferation and decrease the differentiation of growth plate chondrocytes, although this role of IL-6 appears to be controversial [22, 23]. TNF*α* was shown to increase the serum calcium of mice [24] and to stimulate new osteoclast formation and bone resorption [25], whereas it is potently proapoptotic for osteoblasts [26]. Previously, studies on fracture healing after hemorrhagic shock did not focus on the systemic inflammatory response in their models. The chosen durations of shock were relatively short in comparison to most established experimental shock models [27]. This shortened time of depressed blood pressure might result in a lower immunological reaction [28, 29]. Therefore, it might be speculated that the positive effects on fracture healing were mostly related to improved blood flow properties after resuscitation with colloids or crystalloids.

In contrast to the aforementioned studies, Wichmann et al. reported increased osteocyte necrosis adjacent to the fracture site and a decrease of plasma calcitonin levels after hemorrhagic shock when compared to a closed fracture without associated hemorrhagic shock [8]. Therefore, we chose a comparable pressure controlled shock model for our study. As expected, we measured a significant increase in inflammatory cytokines after the shock period comparable to preceding studies [29]. Similar to two studies which used comparable shock models [8, 30], we found an impaired fracture healing response after hemorrhagic shock.

We demonstrated a decrease in bone quality scoring and an increased number of cartilage islands within the callus region three weeks after hemorrhage. This suggests impaired maturation of the callus. This impairment may to some extent be explained by the reduced number of osteoclasts after shock. Osteoclasts are activated during the inflammatory stage of fracture healing. They are responsible for the resorption of damaged and necrotic tissue to prepare the field for the reparative phase [31]. Nevertheless, the initial stage of enchondral fracture repair does not necessarily depend on osteoclasts [32], although they may contribute to vascular invasion and early enchondral ossification [33]. In the further stages of healing, the inhibition of osteoclasts is associated with delayed hard callus remodeling [32]. Therefore, the reduced number of osteoclasts after hemorrhagic shock might be responsible for delayed clearance of necrotic tissue as well as impaired remodeling of callus. This suggestion is in line with another recent study which showed a decreased rate of osteoclasts 8 days after hemorrhagic shock whereas the differentiation of osteoblasts was unaffected [30].

In both histological and *μ*CT analyses, we observed bridging of the fracture site with bone in both groups. This is in line with Manigrasso and O'Connor who reported that callus volume decreases after 14 days [34]. Hemorrhagic shock did not appear to have any effect on BMD or BV/TV within the callus region measured by *μ*CT analyses. However, there was a trend towards decreased TMD in the shock group, indicating that the bone formed in this model may have an inferior quality of mineralization. With a voxel size of 25 *μ*m, the grey value of the bone surface voxels will be subject to partial volume effects. It is therefore difficult to ascertain whether the TMD values observed here are accurate depictions of the state of mineralization of the tissue. Future studies in this area should apply a smaller voxel size (<12 *μ*m) in order to produce a more accurate calculation of TMD.

A crucial aspect of healing is that new tissue in the fracture zone must provide sufficient strength to the injured limb in order to regain function. To investigate the stability of the callus zone, we chose the distraction-to-failure model as a mode of testing because the geometry of the fracture is variable. Despite stabilization of the fracture, this renders more standard biomechanical tests less accurate. The analysis of the mechanical properties of inhomogeneous and porous materials like bone is easily affected by the distribution of the load throughout the specimen [35, 36]. The three point bending test for example has maximum load at only one point of the specimen. To eliminate measurement artifacts caused by variable femur thickness and shape, we chose tensile testing to homogeneously strain the femur cross-section. At three weeks, the maximum force at failure of the HS group was equivalent to the sham group. The decreased failure strength of the fracture zone in comparison to the uninjured bone is consistent with a previous study, which indicated that significant increases in structural or material strength did not occur until 6 to 12 weeks after fracture [34]. In our model, we are not able to report any information about the stiffness of the fracture region. Therefore, stiffness differences between Sham and HS groups are a topic for future studies. Additionally, we performed a selective examination three weeks after fracture, which is during the remodeling phase. Therefore, this time point might have been too delayed to measure significant differences in mechanical stability despite histological evidence of impaired fracture healing. Another study that assessed the influence of simvastatin on fracture healing might support this assumption. Simvastatin-related increases in callus volume could be measured only two weeks after fracture [37]. At three weeks, they noted no differences in mechanical strength or callus volume between the control and drug-treated group. Therefore, potential differences in mechanical properties as well as callus volume and mineralization should be analyzed in further studies at earlier time points.

In conclusion, our results suggest an impaired maturation of the fracture callous three weeks after hemorrhagic shock. Changes in the number of osteoclasts may play an important role in this bone healing delay.

## References

[1] Audigé L., Griffin D., Bhandari M., Kellam J., Rüedi T. P. (2005). Path analysis of factors for delayed healing and nonunion in 416 operatively treated tibial shaft fractures. *Clinical Orthopaedics and Related Research*.

[2] Andermahr J., Elsner A., Brings A. E., Hensler T., Gerbershagen H., Jubel A. (2006). Reduced collagen degradation in polytraumas with traumatic brain injury causes enhanced osteogenesis. *Journal of Neurotrauma*.

[3] Braithwaite R. S., Col N. F., Wong J. B. (2003). Estimating hip fracture morbidity, mortality and costs. *Journal of the American Geriatrics Society*.

[4] Claes L., Recknagel S., Ignatius A. (2012). Fracture healing under healthy and inflammatory conditions. *Nature Reviews Rheumatology*.

[5] Lee S.-K., Lorenzo J. (2006). Cytokines regulating osteoclast formation and function. *Current Opinion in Rheumatology*.

[6] Lucas T. S., Bab I. A., Lian J. B. (1997). Stimulation of systemic bone formation induced by experimental blood loss. *Clinical Orthopaedics and Related Research*.

[7] Bumann M., Henke T., Gerngross H., Claes L., Augat P. (2003). Influence of haemorrhagic shock on fracture healing. *Langenbeck's Archives of Surgery*.

[8] Wichmann M. W., Arnoczky S. P., DeMaso C. M., Ayala A., Chaudry I. H. (1996). Depressed osteoblast activity and increased osteocyte necrosis after closed bone fracture and hemorrhagic shock. *The Journal of Trauma*.

[9] Bonnarens F., Einhorn T. A. (1984). Production of a standard closed fracture in laboratory animal bone. *Journal of Orthopaedic Research*.

[10] Drescher W., Beckmann R., Kasch R. (2011). Nitrate patch prevents steroid-related bone necrosis. *Journal of Orthopaedic Research*.

[11] Augat P., Margevicius K., Simon J., Wolf S., Suger G., Claes L. (1998). Local tissue properties in bone healing: influence of size and stability of the osteotomy gap. *Journal of Orthopaedic Research*.

[12] Yamaji T., Ando K., Wolf S., Augat P., Claes L. (2001). The effect of micromovement on callus formation. *Journal of Orthopaedic Science*.

[13] Wolf D.-I. S., Janousek A., Pfeil J. (1998). The effects of external mechanical stimulation on the healing of diaphyseal osteotomies fixed by flexible external fixation. *Clinical Biomechanics*.

[14] Glowacki J. (1998). Angiogenesis in fracture repair. *Clinical Orthopaedics and Related Research*.

[15] Wallace A. L., Draper E. R. C., Strachan R. K., McCarthy I. D., Hughes S. P. F. (1991). The effect of devascularisation upon early bone healing in dynamic external fixation. *Journal of Bone and Joint Surgery—Series B*.

[16] Starr A. J., Welch R. D., Eastridge B. J., Pierce W., Zhang H. (2002). The effect of hemorrhagic shock in a caprine tibial fracture model. *Journal of Orthopaedic Trauma*.

[17] Stanley K. T., VanDort C., Motyl C., Endres J., Fox D. A. (2006). Immunocompetent properties of human osteoblasts: interactions with T lymphocytes. *Journal of Bone and Mineral Research*.

[18] Lorenzo J., Horowitz M., Choi Y. (2008). Osteoimmunology: interactions of the bone and immune system. *Endocrine Reviews*.

[19] Shen F., Ruddy M. J., Plamondon P., Gaffen S. L. (2005). Cytokines link osteoblasts and inflammation: microarray analysis of interleukin-17- and TNF-*α*-induced genes in bone cells. *Journal of Leukocyte Biology*.

[20] Manolagas S. C., Jilka R. L. (1995). Bone marrow, cytokines, and bone remodeling: emerging insights into the pathophysiology of osteoporosis. *New England Journal of Medicine*.

[21] Roodman G. D. (1992). Interleukin-6: an osteotropic factor?. *Journal of Bone and Mineral Research*.

[22] Kitamura H., Kawata H., Takahashi F., Higuchi Y., Furuichi T., Ohkawa H. (1995). Bone marrow neutrophilia and suppressed bone turnover in human interleukin-6 transgenic mice: a cellular relationship among hematopoietic cells, osteoblasts, and osteoclasts mediated by stromal cells in bone marrow. *The American Journal of Pathology*.

[23] Jikko A., Wakisaka T., Iwamoto M. (1998). Effects of interleukin-6 on proliferation and proteoglycan metabolism in articular chondrocyte cultures. *Cell Biology International*.

[24] Tashjian A. H., Voelkel E. F., Lazzaro M., Goad D., Bosma T., Levine L. (1987). Tumor necrosis factor-*α* (Cachectin) stimulates bone resorption in mouse calvaria via a prostaglandin-mediated mechanism. *Endocrinology*.

[25] Stashenko P., Dewhirst F. E., Peros W. J., Kent R. L., Ago J. M. (1987). Synergistic interactions between interleukin 1, tumor necrosis factor, and lymphotoxin in bone resorption. *Journal of Immunology*.

[26] Jilka R. L., Weinstein R. S., Bellido T., Parfitt A. M., Manolagas S. C. (1998). Osteoblast programmed cell death (apoptosis): modulation by growth factors and cytokines. *Journal of Bone and Mineral Research*.

[27] Hauser C. J. (2005). Preclinical models of traumatic, hemorrhagic shock. *Shock*.

[28] Pfeifer R., Lichte P., Schreiber H. (2013). Models of hemorrhagic shock: differences in the physiological and inflammatory response. *Cytokine*.

[29] Kobbe P., Vodovotz Y., Kaczorowski D. J., Mollen K. P., Billiar T. R., Pape H.-C. (2008). Patterns of cytokine release and evolution of remote organ dysfunction after bilateral femur fracture. *Shock*.

[30] Neunaber C., Yesilkaya P., Pütz C., Krettek C., Hildebrand F. (2013). Differentiation of osteoprogenitor cells is affected by trauma-haemorrhage. *Injury*.

[31] Schindeler A., McDonald M. M., Bokko P., Little D. G. (2008). Bone remodeling during fracture repair: the cellular picture. *Seminars in Cell and Developmental Biology*.

[32] McDonald M. M., Dulai S., Godfrey C., Amanat N., Sztynda T., Little D. G. (2008). Bolus or weekly zoledronic acid administration does not delay endochondral fracture repair but weekly dosing enhances delays in hard callus remodeling. *Bone*.

[33] Deckers M. M. L., Van Beek E. R., Van Der Pluijm G. (2002). Dissociation of angiogenesis and osteoclastogenesis during endochondral bone formation in neonatal mice. *Journal of Bone and Mineral Research*.

[34] Manigrasso M. B., O’Connor J. P. (2004). Characterization of a closed femur fracture model in mice. *Journal of Orthopaedic Trauma*.

[35] Keaveny T. M., Guo E., Wachtel E. F., McMahon T. A., Hayes W. C. (1994). Trabecular bone exhibits fully linear elastic behavior and yields at low strains. *Journal of Biomechanics*.

[36] Kopperdahl D. L., Keaveny T. M. (1998). Yield strain behavior of trabecular bone. *Journal of Biomechanics*.

[37] Skoglund B., Forslund C., Aspenberg P. (2002). Simvastatin improves fracture healing in mice. *Journal of Bone and Mineral Research*.

